# Determination of Efficacy of Reflexology in Managing Patients with Diabetic Neuropathy: A Randomized Controlled Clinical Trial

**DOI:** 10.1155/2014/843036

**Published:** 2014-01-09

**Authors:** Krishna Dalal, V. Bharathi Maran, Ravindra M. Pandey, Manjari Tripathi

**Affiliations:** ^1^Department of Biophysics, All India Institute of Medical Sciences, Ansari Nagar, New Delhi 110 029, India; ^2^Department of Biostatistics, All India Institute of Medical Sciences, Ansari Nagar, New Delhi 110 029, India; ^3^Department of Neurology, All India Institute of Medical Sciences, Ansari Nagar, New Delhi 110 029, India

## Abstract

*Background*. The restricted usage of existing pharmacological methods which do not seem to provide the treatment of diabetic neuropathy may lead to exploring the efficacy of a complementary therapy. In this context, this paper was devoted to evaluate the efficacy of foot reflexology. This health science works on the hypothesis that the dysfunctional states of body parts could be identified by observing certain skin features and be rectified by stimulating certain specific areas mapped on feet. *Method*. Subjects (*N* = 58) with diagnosed diabetic neuropathy were randomly distributed into reflexology and control groups in which both group patients were treated with ongoing pharmacological drugs. Reflexology group patients were additionally treated holistically with the hypothesis that this therapy would bring homeostasis among body organ functions. This was a caregiver-based study with a follow-up period of 6 months. The outcome measures were pain reduction, glycemic control, nerve conductivity, and thermal and vibration sensitivities. The skin features leading to the detection of the abnormal functional states of body parts were also recorded and analyzed. *Results*. Reflexology group showed more improvements in all outcome measures than those of control subjects with statistical significance. *Conclusion*. This study exhibited the efficient utility of reflexology therapy integrated with conventional medicines in managing diabetic neuropathy.

## 1. Introduction

Diabetes is the single most important metabolic disease, which can affect nearly every organ system in the body [1]. The prevalence of diabetes for all age groups worldwide was estimated to be 2.8% in 2000 and was expected to be raised up to 4.4% in 2030. The most important demographic change to diabetes prevalence across the world appears to be increased in the proportion of people older than 65 years of age [2]. According to the Diabetes Atlas 2006 published by the International Diabetes Federation, the number of people with diabetes in India is currently around 40.9 million and is expected to be raised to 69.9 million by 2025 unless urgent preventive steps are taken [3]. Much more alarming is the growing incidences of gestational diabetes in India, of which 60 percent of the population is known to develop diabetes later in life. These statistics are of great public health concern, because people with diabetes are 25 times more likely to develop blindness, 17 times more likely to develop kidney disease, 30 to 40 times more likely to undergo amputation, two to four times more likely to develop myocardial infarction, and twice as likely to suffer from a stroke than nondiabetics [4]. The common complication of diabetic mellitus is diabetic neuropathy. The pain can be severe and effectively destroy the quality of one's life [5]. Development in the different types of neuropathy is correlated with the duration of diabetes and glycemic control. The commonest form of diabetic neuropathy is distal symmetric polyneuropathy in which the patients present with distal sensory loss, loss of ankle reflex, and abnormal position sense. The other features are hyperesthesia, paresthesia, and related pain [6]. Neuropathic pain occurs typically in the lower limb, usually presents at rest and worsens at night. With the progressive neuropathy, the associated pain subsides and eventually disappears and a sensory deficit in the lower extremities persists. The management of such a case is to improve (i) glycemic control, (ii) nerve conduction velocity, and (iii) vibrational and thermal sensitivities. However, with the use of conventional therapy, managing diabetic neuropathy is less than satisfactory [7]. Since the metabolic disorder can affect almost all the organ systems, in order to deal with such a situation, much attention should be paid to rectify the functioning of all the organ systems holistically together with stress management [8]. By adopting this procedure, one can only reverse the symptoms of neuropathy. And to realize this hypothesis for achieving maximum benefits, there has been considerable interest in integrating conventional therapies with “complementary and alternative medicine” (CAM) among the population in a larger part of the globe.

Reflexology therapy has emerged as a form of noninvasive and nonpharmacological complementary therapy for several medical conditions [9, 10]. Reflexology is the science of studying the human health through certain specific reflex/reflexology areas (RAs) quantized on feet, hands, and ears. It is reported in the literature that the application of reflexology therapy may rectify the organ dysfunction and establish homeostasis in the organ function [11, 12]. With its known beneficial effects on the improvement of the quality of life in cancer patients [13], reduction in pain and anxiety for patients with breast and lung cancer [14] and premenstrual syndrome [15, 16], reduction in seizure frequency, and improvement of the quality of life in intractable epilepsy [17], this clinical trial was launched to determine the efficacy of reflexology in the patients suffering from diabetic neuropathy. The hypothesis of reflexology is that the skin areas of feet/hands/ears are the representative of the target body parts and impulses generated on the reflexology areas by the external stimulations of definite intensities arrive at the target body parts through the neural pathways or through hormone-like activities [18]. These impulses are supposed to rectify the corresponding abnormally functioning status of the referred ones, if there is any. The working principle of reflexology therapy may follow the same one as that reported in acupuncture techniques [19, 20]. Keeping this in view, the present trial was conducted based on the aforesaid hypothesis of reflexology that the stimulations generated by the finger movements on foot reflexology areas (RAs) would restore the homeostasis of the body organ functions and hence have therapeutic effect on diabetic neuropathy. In this context, it may be mentioned that the previous studies conducted by the presenting authors could reveal a few characteristic abnormal features of RAs corresponding to certain abnormally functioning body parts [21, 22].

## 2. Methods

The methodologies are detailed in the subsections to follow.

### 2.1. Settings and Location

The subjects were recruited from the outpatient Department of Neurology, All India Institute of Medical Sciences, New Delhi, India, and were referred to the Department of Biophysics of the same institute for applying reflexology therapy. The clinical trial was ethically approved by the institutional ethics review board.

### 2.2. Sample Size Calculation

The sample size of this study was calculated considering reduction in neuropathic pain (measured by using visual analogue scale (VAS) score) as the primary outcome variable. With anticipated mean ± SD of VAS score in control group as 3.4 ± 0.5 [23] and expecting 40% more improvement in reflexology group, the sample size was calculated to be 36 samples per group with 95% confidence level and 90% power.

### 2.3. Subjects Screening and Randomization

**Figure 1:**
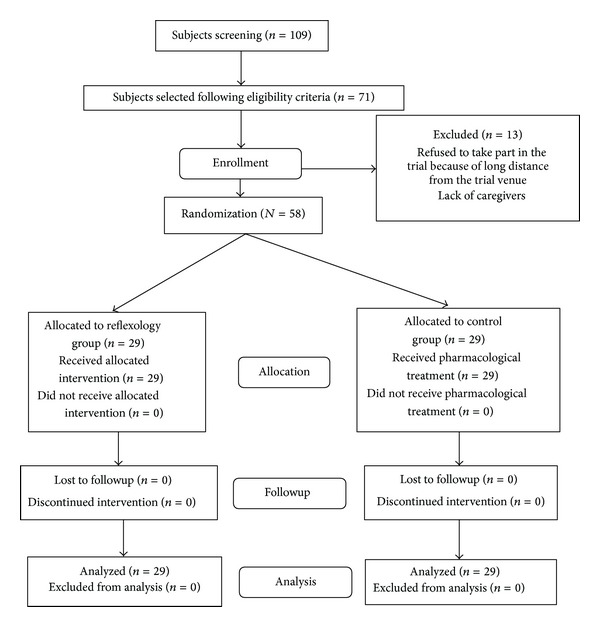
Consort diagram for subjects' recruitment and followup.

A number of 109 subjects were screened to meet with the eligibility criteria of the clinical trial and a sample size of 71 subjects, diagnosed diabetic neuropathy, was selected for recruitment in the trial. A group of 13 patients were excluded from the trial due to their refusal to apply reflexology therapy by their own caregivers and being nonresidents of Delhi territory. Thus the trial ended up with 58 subjects who were allocated blindly to either reflexology group (reflexology therapy + conventional therapy) or to control group (conventional therapy alone) by computer generated random number in block randomization with 4 candidates per block. This procedure of randomization led to the recruitment of 29 subjects in reflexology group and 29 patients in control group, respectively. The details of the study subjects' recruitment procedure are presented in a consort flow diagram as shown in Figure 1.

### 2.4. Eligibility Criteria for Participants

Patients, with documented peripheral diabetic neuropathy whose glycosylated haemoglobin >6.5% and plasma glucose level ≥120 mg/decilitre (fasting) and ≥200 mg/deciliter (postprandial) for more than 1 occasion of any age of both genders, were included in the study. Subjects with end organ damage (namely, gangrene, or toes or foot amputation) due to diabetes mellitus/any cause, chronic disorders like malignancy, tuberculosis, asthma, or any communicable disease were excluded from the study.

### 2.5. Instructions for Both Group Patients

All the subjects were made to understand the importance of their active participations in this study by explaining the known effects and the hypothesis of reflexology therapy. The written instruction sheets, elaborating the therapy procedure as well as the protocol to be followed for their participations in the trial, were distributed to all the participants. It was thus assumed that there would be uniformity in the compliance of both group subjects.

### 2.6. Subjects' Preparation prior to Optical Data Collection

The hypothesis that the functional status of an organ may be detected by observing certain features on the feet was explained to the subjects prior to their participations for recording the externally visible data on the feet. The instructions for maintaining a specific protocol of the pre-data-acquisition procedure were followed for maintaining the identical conditions of feet. The subjects' feet were cleaned by wet cloth and allowed to air-dry while resting for 15 minutes. This procedure enabled one not only to make the feet optically clean but also to establish a uniform blood circulation in the feet. The whole procedure was conducted at an average room temperature of 25°C.

### 2.7. Physical Assessment of the RAs

All the reflexology areas were carefully observed and compared if there were the following abnormal signatures: (i) tenderness under a defined finger pressure as assessed by a pedography system (Emed-AT/2, Novel GmbH, Germany) in the range 30 N/cm^2^ to 35 N/cm^2^, (ii) a definite change in the related skin colour (namely, reddish brown/brown/dark brown/black), (iii) swelling (convex formation), (iv) hollowness (concave formation), (v) the feeling of depression with a finger pressure of a well defined strength, and (vi) the feeling of the presence of tiny granules with blurred finger nail scratching. The presence of any or a combination of these features on a RA led to conclude the area to be “abnormal” and hence the referred organ was declared to be abnormal [22]. The localized tenderness on a RA in response to the defined finger pressure was measured with the help of a VAS score with the scale range of “0” to “10.”

### 2.8. Optical and Swept Source-Optical Coherence Tomography (SS-OCT) Image Data Recordings

The skin images of the RAs were recorded, during the pretherapy sessions under the normalized condition as described, using an optical camera (Nikon D200, Japan). The SS-OCT with dermatology applications (OCM1300SS, Thorlabs Incorporated, Newton, New Jersey), comprising a high-speed frequency-swept external cavity laser (*λ*_central_ = 1325 nm), 3-dB spectral bandwidth (>100 nm), and an average output power of 10 mW, was used to record the subcutaneous data related to urinary bladder RAs at the pretherapy session. The instrument used for this purpose was described in detail in previous publications [21, 24].

### 2.9. Study Design for Reflexology Group Patients

A foot reflexology therapy application protocol was developed to treat the patients holistically. A step-by-step procedure was followed uniformly to stimulate the following RAs: energy balance [25], lymphatic system, solar plexus [26], adrenal glands, spine, urinary system, digestive system, brain, other endocrine glands, sciatic nerve, knee and hip. The hypothesis of this protocol was that stimulations on these specific areas would establish homeostasis in the functional status of the lymphatic, urinary, digestive and immunity systems together with releasing the mental stress, improving the diabetic control [27] and increasing the lower limb activities. The maps of the reflexology areas for these target body parts located on the feet had been followed as per the standard descriptions of various publications [28–30], Indian school of reflexology practice [31], and in-house data records.

The patients were asked to visit the reflexology laboratory once in a week during the first month of the trial as this was the period for generating trained caregivers. During this period, the performances of the caregivers in applying therapy to their patients were checked every week. The patients' responses to a particular therapy step were recorded to note the effect of stimulations on the corresponding reflexology areas of the referring system.

Stimulations in the form of mechanical pressure and relaxation on a particular RA were uniformly produced by fingers using both the hands of the caregivers. The fingers of one hand were used for producing stimulations and the other one for holding the foot firmly against the application of pressure. Moderate pressure, in the range of 30 N/cm^2^ to 35 N/cm^2^ as recorded using pedography system, Emed-AT/2, Novel GmbH (Germany) [32], with tolerable tenderness was used to generate stimulations. The areas were lubricated with cream of milk without any additive before applying stimulation in order to avoid any adverse effect on the skin due to friction. Each reflexology area was stimulated (average) 15 times of ~20 seconds duration per session with the understanding that stimulations on a particular RA < 10 times did not produce any therapeutical effect and a RA would be overstimulated with continuous stimulations >20 times. One therapy session took ~1/2 hour duration and there were 2 therapy sessions per day.

### 2.10. Conventional Therapy for Both Groups

The institute standard mode of pharmacological management for treating neuropathy with diabetic mellitus was followed for both groups during the trial period.

### 2.11. Evaluation of Treatment Compliance for Both Groups during Follow-Up Period

For the purpose of followup, the subjects were present at the reflexology laboratory (for reflexology group) and neuropathy clinic (both groups) once in a month for the remaining five months (initial one month was the training period for reflexology group patients). The quality assurance of the compliance of both group patients was monitored critically by pill counting method [33] (both groups) and by the reflexology method [21, 22] (reflexology group) as well as by noticing the individual performances of the caregivers while applying the therapy. An identical method of health monitoring program for both the groups was followed and it was independently done by the clinicians physically located separately from the reflexology laboratory. The follow-up period was 6 months including the training period (reflexology group). There was an option to the participating subjects for withdrawing themselves from the trial at their own desire. However, the trial did not encounter any such situation.

### 2.12. Outcome Measures

The primary outcome measure was neuropathic pain using VAS score and the secondary outcome measures were the levels of HbA1c and blood glucose (fasting and postprandial), thermal and vibration sensitivity, nerve conduction velocity (NCV), and quality of life. The quality of life was evaluated with the help of “neuroQoL” instrumentation [34] to assess the following: (1) symptomatic affliction, (2) psychosocial impairment, (3) diabetic neuropathy-specific impact, and (4) overall quality of life. The mode of measuring the quality of life using NeuroQoL was that the lower was the score, the better was the quality of life [35]. Thermal and vibration sensitivities were measured by using Vibrotherm analyzer (Vibrotherm Dx, Diabetik Foot care, India) [35, 36]. As per the data supplied by the manufacturer, the thermal (both hot and cold) perception threshold was measured in the temperature range of 1°C to 50°C with the rate of temperature change of 1°C per second. These were recorded by keeping a constant contact of a hand held probe tip of diameter 20 mm to the specific areas on the feet. The test was performed at an average room temperature of 25°C. The perception of the probe temperature more than 32°C was considered to be “abnormal” hot sensation and that less than 23°C was taken as “abnormal” cold sensation. The vibration perception threshold was assessed using the vibration probe tip of 15 mm diameter and vibration frequency of 100 Hz in the amplitude range of 0–50 volts. The specified normal threshold vibration amplitude was ≤15 volts.

Peripheral nerve conduction studies were performed using Viking Quest, Nicolet Viasys Healthcare, (Nicolet Biomedical, USA) [37–39]. Both motor and sensory nerves were examined using the standard settings and filters as specified by the manufacturer. The appropriate anatomical sites were stimulated using a surface electrical stimulator. The normative data of the parameters were used from the study institute reference values.

### 2.13. Data Analyses

Data were presented as number (%) or mean ± SD or median (range) as appropriate. In this trial, age and neuropathic pain score (VAS) was found to be normally distributed. Baseline characteristics were summarized between the groups. The median (range) for % improvements in neuropathy (VAS score) in the two-groups and the difference in % improvements were compared using two group Wilcoxon rank sum test (nonparametric test). Difference in means between the groups was compared using Student's *t*-test for independent samples. The change in posttherapy values from baseline was tested for continuous variables using paired *t*-test and categorical variables using Mc Nemar's statistical analysis. The differences in proportions between the groups were tested using Chi square test/Fisher's exact test. The *P* value less than 0.05 was considered statistically significant. Statistical analysis was carried out using STATA 9.0 (College station, Texas, USA).

The % improvement in neuropathy of an individual subject within a group was calculated in terms of % reduction in pain and was defined according to (1) as follows:

(1) %  reduction in neuropathy  =(VASpre-th−VASpost-th)VASpre-th×100.

VAS_pre-th_ and VAS_post-th_ are the pain scores at the baseline and at the end of follow-up period, respectively. The positive or negative sign of (1) indicates reduction or enhancement in neuropathic pain at the posttherapy session with respect to the baseline one.

Similarly, the % improvement within a group in either of the specified parameters like HbA1c and Blood glucose (fasting and post prandial) in which the levels are supposed to be reduced with therapy, if the same would have positive response, was also determined using the following equation, similar to (1):

(2) %  improvement in parameter  =(Parameterpre-th−Parameterpost-th)Parameterpre-th×100.

Parameter_pre-th_ and Parameter_post-th_ represent the mean value of either of the following: HbA1c or fasting blood glucose and postprandial blood glucose, at the pretherapy session and posttherapy session, respectively, within a specified group.

## 3. Results

The results of this clinical trial are detailed in the subsections to follow.

### 3.1. Demographics

All the data are presented in terms of (mean ± SD) or median (range) in this section. The age (mean ± SD), gender ratio (male : female), the duration of diabetes mellitus [median (range)] and the duration of neuropathy [median (range)], for reflexology group (*n* = 29) were 56.8 ± 9.7 years; 16 : 13; 10 (3–28) years; 5 (1–14) years, respectively. The respective values for control group (*n* = 29) were 55.9 ± 11.2; 15 : 14; 13 (4–30); 6 (2–8). All the baseline data were statistically comparable between two groups, except the abnormal perception of cold and vibration sensitivities.

### 3.2. Observations on Primary Outcome Measure

The baseline VAS score, describing pain intensity, was reduced from 7.8 ± 1.4 to 3.0 ± 1.8 at the end of follow-up period with highly statistical significance (*P* value <0.001) for reflexology group patients. Control group subjects exhibited VAS score reduction from baseline 7.0 ± 1.4 to 6.0 ± 1.7 at the end of follow-up period. The median [range] improvement in reflexology group was 66.6% [14.2%–90.0%] and that in control group was 14.2% [(−25%)–87.5%]. That is, difference in neuropathic pain reduction in reflexology group with respect to that of control group patients was found to be 52.4% with a *P* value <0.001.

### 3.3. Observations on Secondary Outcome Measures

The data on secondary outcome measures are tabulated in Tables 1 and 2 which exhibit that there was positive response in each category of the parameters with statistical significance (*P* < 0.05) among reflexology group patients. The maximum response was obtained in improving cold sensitivity with 100% response among reflexology group subjects. Each of the responses was found to be statistically significant except postprandial blood glucose frequency (*P* = 0.082).

The quality of life in patients with diabetic neuropathy, measured by NeuroQoL instrument, revealed that the baseline score was reduced from 8 (4–10) to 4 (1–6) at the end of follow-up period in reflexology group, whereas in control group it was reduced from 7 (4–9) to 5 (3–8). This result reveals that there was 21.3% more improvement in reflexology group than that of control one with *P* value = 0.001 and 95% confidence interval.

### 3.4. Observation on Reflexology Areas

**Figure 2:**
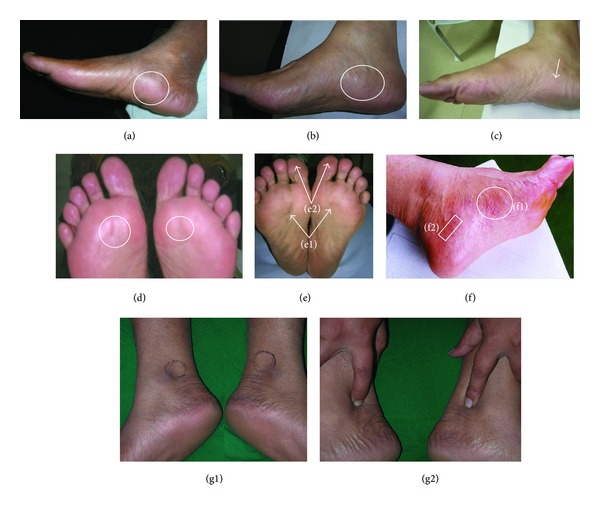
Abnormal skin features of the reflexology areas (RAs) of urinary bladder (UB), solar plexus, pituitary gland, pancreas, lumbar vertebrae, and sciatic nerve. (a)–(c) convex UB RAs; (a) 66M; (b) 73M; (c) 53F convex and reddish UB RA; (d) 65F: reddish brown and concave solar plexus RAs; (e) 56F: dark brown and concave solar plexus RAs (e1) and dark brown pituitary gland RAs (e2); (f) 58M: reddish brown pancreas RA (f1) and reddish brown lumbar vertebrae RA (f2); (g1)-(g2) 30F: concave and brown sciatic nerve RAs.

**Figure 3:**
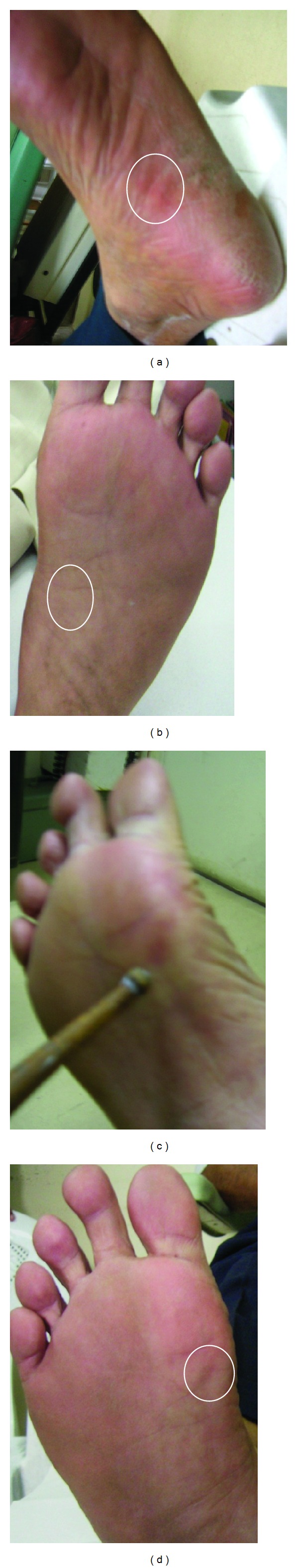
Observations on pancreas (63M) and adrenal gland (58M) reflexology areas at the pre- and post-reflexology therapy sessions. (a) pretherapy session: reddish brown skin colour of pancreas RA; (b) posttherapy session: pancreas RA with normal skin colour; (c) pretherapy session: reddish brown adrenal gland RA and (d) posttherapy session: adrenal gland RA with normal skin colour.

**Figure 4:**
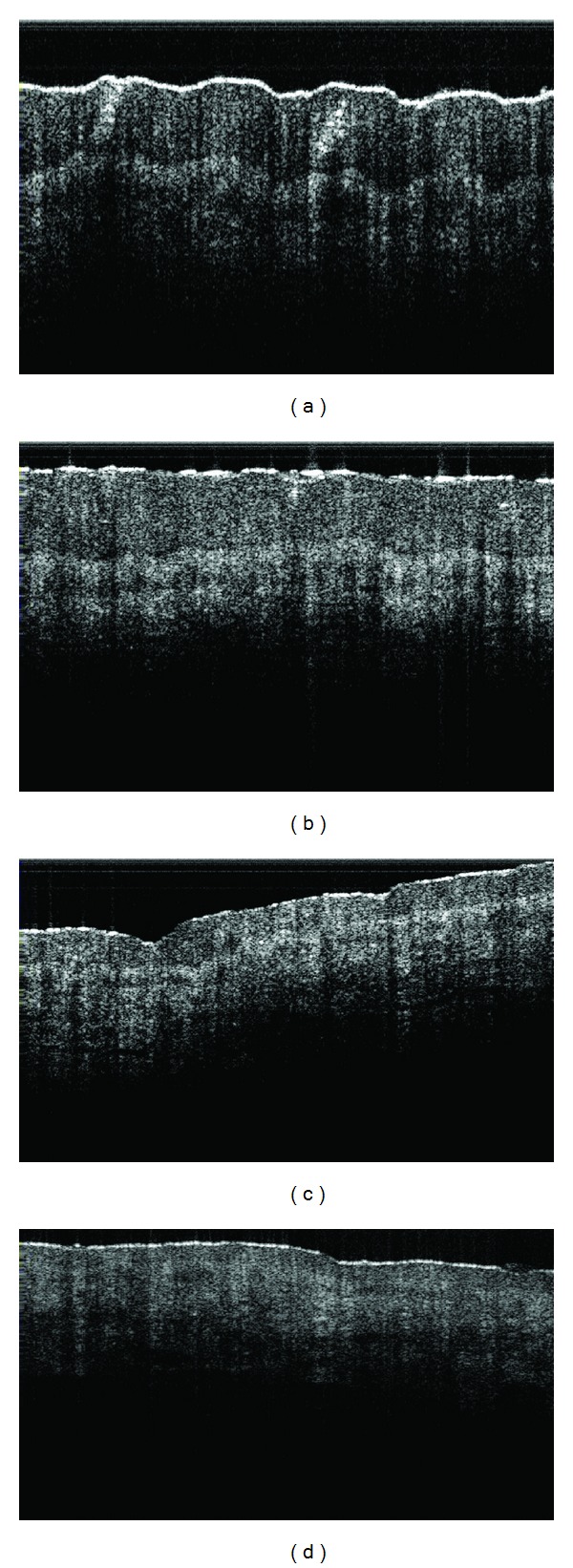
A few examples of the subcutaneous features (up to 1.75 mm) of urinary bladder reflexology areas. (a) A normal structure (without the presence of any abnormal skin characteristics); (b) the onset of an abnormal condition (tender RA); (c) an abnormal condition (tender and swollen RA); (d) an advanced stage of abnormality (tender, swollen and hard skin).

The abnormal physical characteristics like tenderness, hyperpigmentation, convexity (swelling), concavity (hollowness formation), change in skin colour, and the feeling of the presence of tiny granules in RAs were noted among reflexology group patients. A few examples on abnormal RAs are shown in Figure 2. These externally observed features of different RAs got improved with statistical significance (*P* value = 0.001) at the end of the follow-up period (Table 3).  Figure 3 exhibits 2 examples of abnormal skin colour at the baseline and the respective status at the end of follow-up period. Abnormal reddish brown skin colour of pancreas and that of adrenal gland RAs disappeared with reflexology therapy and these RAs exhibited normal skin colour at the end of the follow-up period.  Figure 4 depicts the subcutaneous (up to 1.75 mm) features of the urinary bladder RAs. It describes the subcutaneous layer characteristics related to “normal” and different categories of “abnormal” RAs.

## 4. Discussion

This was an open label clinical trial based on the assumption that reflexology therapy intervention would achieve 40% improvement from the result with ongoing pharmacological drugs and accordingly the required sample size was estimated. Improvement in the primary outcome measure, that is, on the reduction of neuropathic pain, in reflexology group at the end of follow-up period with respect to the baseline was observed to be 52.4% more than control group. As the difference in primary outcome variable was statistically significant (*P* value <0.001) and more than the anticipated one, this study ending with 29 subjects per group had adequate power to detect the observed difference.

All the subjects were admitted into the trial as per the selection criteria and were randomized blindly irrespective of age, gender, socioeconomic status, and duration of neuropathy. Much attention was paid amongst reflexology group patients for maintaining the quality and uniformity of standardized therapy compliance. However, there could be some variations in reflexology intervention received by the patients through their caregivers performing therapy at their preferable places. This was the main limitation of the trial with reflexology group patients in applying reflexology therapy lacking in quantification because of its manual and subjective nature. The corresponding error was minimized by monitoring the performances of the caregivers on a regular time interval and the quality assurance of therapy compliance was monitored by noting the status of the reflexology areas in terms of tenderness, concavity/convexity, pigmentation, and feeling the presence of foreign tiny particles as demonstrated in Figures 2 and 3.

On completion of the trial, the clinical status of all the study subjects was compared between 2 groups. It was observed that reflexology group patients responded with highly statistical significance. These patients were applied therapy holistically and accordingly, the uniform therapy application protocol was designed and followed. An objective method of monitoring the quality assurance of reflexology therapy compliance was adopted. This method was involved in observing the externally visible abnormal features [21, 24] of the reflexology areas as mentioned. With improvements in different parameters presented in Tables 1 and 2, the abnormal features of the respective RAs also disappeared as supported by the data stated in Table 3. This procedure of evaluating the reflexology areas may offer a method of testing the hypothesis of the reflexology principle that stimulations at the reflexology areas may repair the respective abnormal status and hence rectify the corresponding organ function.

In 2 cases of reflexology group there was no abnormal feature observed on the specified RAs though these subjects were diagnosed with confirmed diabetic neuropathy. At the initial stage of therapy session these patients did not complain of tenderness against moderate finger pressure stimulations applied to any RA in contrast to other 27 reflexology subjects. Later on (after a week) tenderness was felt against the specified finger pressure stimulations and they were treated by the method as followed in other cases. The control group subjects did not take part in the reflexology method of detecting the functional status of the organs and hence the respective characteristics of the RAs were not evaluated.

It may be recalled that all the reflexology group subjects were suffering for 10 years median duration of uncontrolled diabetic mellitus and 5 years median duration of neuropathy and were on the ongoing conventional medicines. In this respect, the holistic application of reflexology therapy in conjunction with the pharmacological drugs improved the blood glucose levels and glycemic control with highly statistical significances. And in its own turn, it would perhaps also pave the way to have remedy to neuropathic pain together with improvement in nerve conduction velocity and thermal and vibration sensitivities as well as in the quality of life. However, though this study indicates the high efficacy of reflexology therapy as an adjunctive therapy to conventional medicines in managing diabetic neuropathy and its complicacy, it is to remember that its application is very much restricted because of its subjective nature.

## 5. Conclusions

This study demonstrated that reflexology therapy in addition to pharmacological therapy may be recommended in reducing the neuropathic pain and improving quality of life and may achieve holistic benefits to the patients suffering from diabetic neuropathy. However, the evaluation of reflexology therapy as an adjunctive regimen warrants further investigations to be carried out in a larger sample size amongst various communities.

## Figures and Tables

**Table 1:** Comparison of pre- and posttherapy glycosylated hemoglobin and blood glucose in between the groups.

Variables	Groups	Frequency of symptom present in samples*		Pre- and posttherapy data comparison (mean ± SD)			
		Pretherapy *n* (*n*%)	Posttherapy *n* (*n*%)	Pretherapy (*n* = 29)	Posttherapy (*n* = 29)	Improvement (%) (2)	*P* value
HbA1c (%)	Reflexology	29 (100%)	10 (34.4%)	9.7 ± 2.5	6.4 ± 1.0	34.0	0.001
	Control	29 (100%)	19 (65.5%)	9.4 ± 1.7	8.6 ± 2.1	8.5	0.001
	*P* value	1.00	0.018	0.5541	0.001		

Fasting blood glucose (mg/dL)	Reflexology	29 (100%)	9 (31.0%)	160.2 ± 46.7	109.6 ± 24.0	31.6	0.001
	Control	29 (100%)	18 (62.1%)	153.4 ± 32.6	130.7 ± 29.5	14.8	0.001
	*P* value	1.00	0.018	0.525	0.012		

Postprandial blood glucose (mg/dL)	Reflexology	29 (100%)	17 (58.6%)	230.0 ± 53.4	141.0 ± 15.8	38.7	0.001
	Control	29 (100%)	24 (82.8%)	220.8 ± 41.9	178.7 ± 40.0	19.1	0.007
	*P* value	1.00	0.082	0.201	0.002		

*The frequency percentage was used to determine the frequency of the trial population presented with a particular physiological parameter.

**Table 2:** Comparison of different parameters between groups.

Observed abnormality in parameters	Groups	Frequency of abnormal parameters present in samples	
		Pretherapy session *n* (*n*%)	Posttherapy session *n* (*n*%)
Perception of thermal (hot) sensation	Reflexology	14 (48.3)	1 (3.4)
	Control	17 (58.6)	13 (44.8)
	*P* value	0.430	<0.001

Perception of thermal (cold) sensation	Reflexology	10 (34.48)	0 (0.00)
	Control	18 (62.07)	14 (48.27)
	*P* value	0.036	<0.001

Perception of vibration sensitivity	Reflexology	13 (44.82)	2 (6.90)
	Control	23 (79.31)	22 (75.86)
	*P* value	0.007	<0.001

Low nerve conduction velocity (NCV)	Reflexology	21 (72.41)	7 (24.13)
	Control	20 (68.96)	21 (72.41)
	*P* value	0.122	0.002

**Table 3:** Comparison of the status of reflexology areas** at baseline and at end of follow-up period (reflexology group: *N* = 29).

Associated clinical symptoms recorded	Status of abnormal reflexology areas (RAs) (abnormal features*)	Frequency (percentage)		* P* value
		Baseline data *n* (%)	Follow-up data *n* (%)	
Subjective poor energy level, loss of self-confidence, frustration, inability to perform paid work, inability to perform daily tasks, and so forth (assessed through neuroQoL instrument [34, 35])	**Energy balance** (tenderness) abnormality			
	(present)	27 (93.10)	3 (10.34)	0.001
	(not present)	2 ( 6.90)	26 (89.66)	

Swollen feet	**Lymphatic system** (tenderness) abnormality			
	(present)	25 (86.21)	4 (13.79)	0.001
	(not present)	4 (13.79)	25 (86.21)	

Stress, anger, worry, depression, maniac, anxiety, restlessness, nervousness	**Solar plexus** (concavity, tenderness, change in skin colour) abnormality			
	(present)	25 (86.21)	7 (24.14)	0.001
	(not present)	4 (13.79)	22 (75.86)	

Low back pain	**Lumbar vertebrae** (concavity, tenderness, change in skin colour) abnormality			
	(present)	21 (72.41)	6 (20.69)	0.001
	(not present)	8 (27.59)	23 (79.39)	

Abnormal nocturia, micturition, syncope while coughing, coughing, sneezing, burning sensation during urination	**Urinary bladder** (convexity, tenderness, change in skin colour) abnormality			
	(present)	24 (82.76)	7 (24.14)	0.001
	(not present)	5 (17.24)	22 (75.86)	

Nausea and vomiting, dyspepsia, constipation, lack of appetite, sour belching, indigestion [40]	**Stomach** (tenderness, feeling of the presence of tiny granules) abnormality			
	(present)	20 (68.96)	7 (24.14)	0.001
	(not present)	9 (31.03)	22 (75.86)	

Sleep disturbance: monophasic biphasic polyphasic	**Brain** (tenderness, feeling of the presence of tiny granules, change in skin colour) abnormality			
	(present)	17 (58.62)	9 (31.03)	0.063
	(not present)	12 (41.38)	20 (68.96)	

Poor glycemic control abnormal blood glucose (fasting and postprandial)	**Pancreas** (tenderness, feeling of the presence of tiny granules, change in skin colour) abnormality			
	(present)	27 (93.10)	6 (20.69)	0.027
	(not present)	2 ( 6.90)	23 (79.39)	

Pricking sensation like needles and pins, shooting and stabbing pain, throbbing sensation in legs, unsteadiness while standing and walking and so forth (assessed through neuroQoL instrument [34, 35])	**Sciatic nerve** (concavity, tenderness, change in colour) abnormality			
	(present)	21 (72.41)	5 (17.24)	0.001
	(not present)	8 (27.58)	24 (82.76)	

*Abnormal features, as mentioned against each RA in this table, were present either alone or in combination.

**Two patients (61 yrs and 65 yrs, duration of diabetics Mellitus > 10 yrs and neuropathy duration > 5 yrs) did not show any abnormally visible features on the foot reflexology areas.
